# Atypical Pattern of Leukocytoclastic Vasculitis in Granulomatosis With Polyangiitis

**DOI:** 10.7759/cureus.49056

**Published:** 2023-11-19

**Authors:** Sarah A Mullen, Julia B Griffin, Allison Lloyd-McLennan, Alfredo Siller, Megan Arthur, Corey J Georgesen

**Affiliations:** 1 College of Medicine, University of Nebraska Medical Center, Omaha, USA; 2 College of Medicine, Creighton University School of Medicine, Omaha, USA; 3 Dermatology, University of Nebraska Medical Center, Omaha, USA

**Keywords:** palpable purpura, leukocytoclastic vasculitis, antineutrophil cytoplasmic antibody (anca) associated vasculitis (aav), granulomatosis with polyangiitis (gpa), -dermatopathology, complex medical dermatology

## Abstract

Granulomatosis with polyangiitis (GPA), formerly Wegener’s granulomatosis, is a small- and medium-vessel vasculitis with characteristic cutaneous morphologic presentation and systemic involvement. Most patients have palpable purpura at some point in their disease course, but this is not always the presenting manifestation. This autoimmune disorder can affect a range of organs, with the upper and lower respiratory tract, kidneys, and nervous system being commonly implicated, while gastrointestinal and cardiac involvement is less frequent. This is a 44-year-old female presenting to the emergency department (ED) with polyarthralgia and palpable purpura. Palpable purpura was distributed on the oral palate, elbow, and lower back, and a punch biopsy revealed leukocytoclastic vasculitis (LCV). While this was an atypical distribution for leukocytoclastic vasculitis, the skin biopsy provided the necessary evidence to diagnose GPA. This case characterizes non-specific and atypical signs and symptoms of GPA that all providers should be aware of in order to diagnose the condition early in its disease course.

## Introduction

Granulomatosis with polyangiitis (GPA), formerly Wegener’s granulomatosis, is diagnosed based on a constellation of clinical findings, histopathology, and specific antibody serology. In the case presented here, the patient presented with a unique distribution of painless purpuric lesions on the oral palate, elbow, and lower back. Punch biopsy of the palpable purpura revealed leukocytoclastic vasculitis (LCV), and serum studies were cytoplasmic antineutrophilic cytoplasmic autoantibody (c-ANCA) and proteinase 3 (PR3) positive. The patient was managed with glucocorticoids and rituximab, with the later addition of azathioprine.

## Case presentation

**Figure 1:**
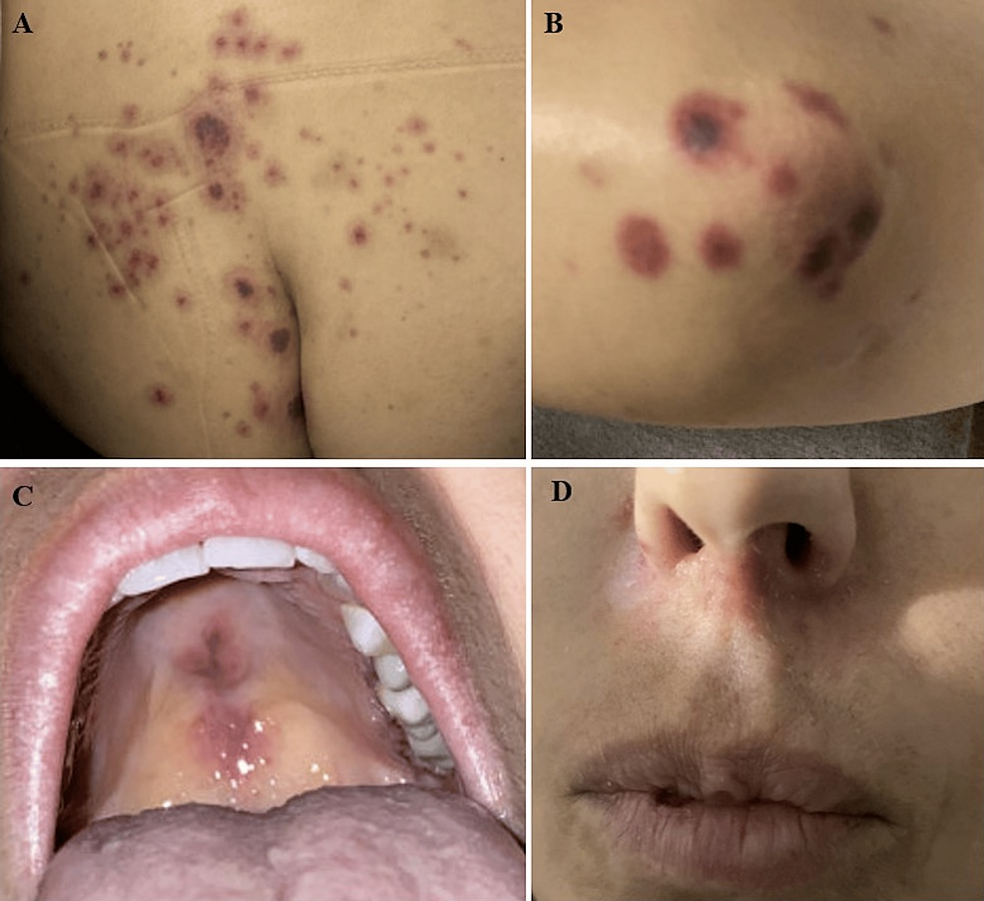
Palpable purpura with a dusky center on the lower back (A) and left elbow (B). Purpuric macules on the oral palate (C) and perinasal crusting (D).

A 44-year-old female presented to the emergency department (ED) with severe joint pain for five months and painless palpable purpura of the left elbow and lower back for one day. Over the prior ten months, she experienced a multitude of symptoms, each attributed to a separate, plausible cause. She had sought care thrice in the ED for an intractable headache and right foot drop; with allergy for worsening of her asthma symptoms, including shortness of breath; and with ophthalmology for periorbital edema and red eyes. She was referred to rheumatology for the migrating polyarthralgia, but she had not been evaluated yet. She had extensive evaluations of these symptoms by different teams, including negative antinuclear antibody (ANA), rheumatoid factor (RF), and ANCA. Her primary care provider was seeing her on an ongoing basis for the management of progressive fatigue, weight loss, and the unresolved symptoms mentioned above. Upon the most recent presentation to the ED, she endorsed onset in the last few days of a Globus sensation, a mildly muffled voice, abdominal pain, diarrhea, nausea, vomiting, and left-hand numbness. On examination, there were palpable purpuric lesions with a dusky center on the lower back and elbow (Figure 1A-1B, respectively). Further, there were similar lesions of the oral palate and perinasal crusting (Figure 1C-1D, respectively). The right eye was red. Almost all joints had pain on palpation. The physical exam was otherwise unremarkable.

**Figure 2:**
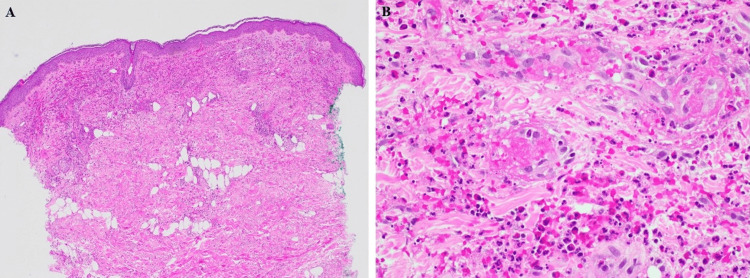
(A) Low power (10×) revealing leukocytoclasis and erythrocyte extravasation; (B) high power (40×) demonstrating dermal fibrinoid necrosis of the small vessels with surrounding neutrophils, eosinophils, and lymphocytes. Direct immunofluorescence was IgA negative.

Laboratory evaluation included but was not limited to elevated creatinine (1.05 mg per dl), hematuria and proteinuria on urine analysis, elevated C-reactive protein and erythrocyte sedimentation rate, low complement C4, positive ANA with 1:40 titer, elevated RF, positive c-ANCA with 1:160 titer, and positive PR-3. A punch biopsy of a purpuric lesion on the lower back revealed vasculitis of the small vessels with mild to medium vessel involvement (Figure 2). A renal biopsy revealed necrotizing glomerulonephritis. Otolaryngology performed a flexible laryngoscopy, showing uvulitis. Neurology obtained nerve conduction studies and electromyography to evaluate for mononeuritis multiplex, which were normal. Ophthalmology evaluation revealed no active uveitis, scleritis, or episcleritis. Based on these findings, she was diagnosed with GPA. Treatment included an extended steroid taper starting at 1 mg/kg intravenous methylprednisolone (IVMP) and transitioning to oral prednisone 60 mg and rituximab. She was treated with rituximab as two 1 mg loading doses two weeks apart for induction of remission, then planned for rituximab 500 mg every six months for maintenance.

## Discussion

While the classic cutaneous pattern of causes of LCV, such as GPA, is purpuric lesions on the upper and lower extremities, this case presents unique locations, including the oral palate, elbow, and lower back. The differential diagnoses for this patient's cutaneous findings also included immunoglobulin A (IgA) vasculitis, other ANCA-associated small-vessel vasculitides, another cause of LCV such as infection, and coagulopathy. While most patients have skin findings at some point in this disease, only about 13% of patients have cutaneous manifestations at initial presentation as painless or painful palpable purpura or petechiae [1]. Therefore, suspicion for this diagnosis must remain high even prior to the presentation of cutaneous findings. The patient also had uvulitis, which, to the best of our knowledge, has not previously been mentioned in association with GPA, although nasal and oropharyngeal symptoms are common [2]. She was also ANCA-negative when first tested. While ANCA-negative GPA is under 10% of clinical cohorts [3,4], up to 17% of GPA patients with limited disease may be ANCA-negative [5]. A 2017 international consensus on ANCA testing recommends high-quality antigen-specific immunoassays for screening and a second PR3-MPO-ANCA or indirect immunofluorescence assay if there is high clinical suspicion but negative initial results [6]. This case presents several initial symptoms that were non-specific manifestations of GPA, highlighting the importance of a broad differential diagnosis and thorough work-up to achieve an early diagnosis. Clinicians must be watchful for other signs and symptoms exemplified in this case, including myalgias, arthralgias, fatigue, and systemic involvement such as ocular involvement [1,7].

This patient required intravenous, higher-potency glucocorticoid therapy with 1 mg/kg IVMP because of her organ-threatening renal disease, whereas patients with less severe disease can be treated with 0.5 mg/kg prednisone as part of the induction regimen [8]. While induction of remission was previously typically achieved with glucocorticoids and cyclophosphamides, randomized controlled trials have since shown induction with rituximab and glucocorticoids to be non-inferior [9,10]. While rituximab is the gold standard for remission therapy, the ideal dosing and regimen are still being investigated [8]. This patient initially improved with rituximab but was unable to taper off prednisone due to the recurrence of red eyes or shortness of breath each time a prednisone taper dose was below 40 mg. After six months of intermittently recurring symptoms, azathioprine 50 mg was added to her regimen as a steroid-sparing therapy alongside rituximab and prednisone 40 mg. One expert consensus states that if remission is not achieved on induction therapy or relapse cannot be controlled with repeated induction therapy, consider options for the use of additional or alternative treatment and consult or refer to a center with vasculitis expertise [11].

## Conclusions

This patient's diagnosis of GPA was delayed multiple months after the presentation of non-specific respiratory, ocular, neurologic, musculoskeletal, and generalized systemic symptoms. Early in the case, the ANCA-PR3-MPO assay was negative. Skin findings presented late in the disease course, including an atypical clinical pattern of leukocytoclastic vasculitis, were a harbinger of severe underlying systemic involvement. This case highlights the need for high clinical suspicion for GPA despite non-specific or atypical findings in order to avoid severe, life-threatening disease manifestations requiring multiple therapies.
